# MDMA-assisted psychotherapy; Inclusion of transgender and gender diverse people in the frontiers of PTSD treatment trials

**DOI:** 10.3389/fpsyt.2022.932605

**Published:** 2022-10-10

**Authors:** Christopher S. Stauffer, Melanie R. Brown, Dee Adams, Marca Cassity, Jae Sevelius

**Affiliations:** ^1^Social Neuroscience and Psychotherapy Lab, Oregon Health and Science Institute, Department of Psychiatry, Portland, OR, United States; ^2^Portland VA Health Care System, Department of Mental Health, Portland, OR, United States; ^3^School of Public Health, Oregon Health and Science University-Portland State University, Portland, OR, United States; ^4^Center for Public Health and Human Rights, Department of Epidemiology, John Hopkins University, Baltimore, MD, United States; ^5^Center of Excellence for Transgender Health, Department of Medicine, University of California, San Francisco, San Francisco, CA, United States

**Keywords:** gender identity (MeSH), transgender persons (MeSH), N-methyl-3, 4-methylenedioxymethamphetamine (MeSH), hallucinogens (MeSH), post-traumatic stress disorder (MeSH), health equity (MeSH), focus groups (MeSH), psychotherapy (MeSH)

## Abstract

**Introduction:**

Transgender and gender diverse (TGD) people experience stigma, discrimination, trauma, and post-traumatic stress disorder (PTSD) at higher rates compared to the general population; however, TGD people have been underrepresented in PTSD research. Clinical trials of 3,4-methylenedioxymethamphetamine (MDMA)-assisted psychotherapy demonstrate promising safety and efficacy for the treatment of PTSD. Issues related to equitable access, power imbalances in the therapeutic relationship, and vulnerable states of consciousness occasioned by MDMA are magnified when working with people affected by structural vulnerabilities and health disparities, and community engagement in research planning and implementation is essential. To inform the inclusion and safety of TGD people in future MDMA-assisted psychotherapy research, the aims of the current study were to: characterize TGD experiences with trauma-related mental health care, assess openness of TGD people to participate in experimental PTSD research, and to gather specific feedback on protocol design for conducting MDMA-assisted psychotherapy with TGD people.

**Materials and methods:**

We conducted three virtual focus group discussions (FGDs) with 5–6 participants each (*N* = 17). Eligible TGD participants had a history of receiving trauma-related mental health care. Each FGD was facilitated by two licensed clinicians who identified as TGD. Qualitative data analysis was conducted via an iterative process of identification of recurrent patterns and themes.

**Results:**

We have identified several key issues TGD people face when seeking and engaging in trauma-related mental health care, including barriers to receiving adequate gender-affirming and trauma-informed mental health care and frustration with providers lacking cultural humility. Suggested amendments to MDMA-assisted psychotherapy protocols include: routine collection of trans-inclusive gender identity data, implementing an explicit gender-affirming treatment approach, ensuring a culturally safe setting, and diversifying co-therapy dyads.

**Discussion:**

The inclusion of TGD voices in early conversations about emerging experimental PTSD interventions promotes equitable access, in the context of health and healthcare disparities, and helps researchers understand the needs of the community and tailor research to meet those needs. Through an ongoing conversation with the TGD community, we aim to incorporate a gender-affirming approach into existing research protocols and inform future applications of MDMA-assisted psychotherapy in addressing the effects of minority stress and boosting resilience.

## Introduction

Transgender and gender diverse (TGD) people experience violence and trauma exposure—and subsequent post-traumatic stress disorder (PTSD) and suicidality—at higher rates than the general population (1, 2). Transgender and gender diverse people are the target of significant experiences of discrimination, including in healthcare settings by healthcare professionals (3). Transgender and gender diverse identities have historically been pathologized within Western medicine. “Gender identity disorder” was removed from the fifth edition of the *Diagnostic and Statistical Manual of Mental Disorders (DSM-5)* in 2013 and replaced with “gender dysphoria.” This, and other positive shifts in terminology in the most recent *DSM-5-TR* (e.g., “desired gender” to “experienced gender”), aim to help destigmatize TGD identities while maintaining healthcare access for those who need it (4, 5). Nonetheless, a recent surge in anti-transgender legislation continues to contribute to the structural vulnerability of TGD people (6–8).

Throughout recorded history, hundreds of distinct cultures have recognized third, fourth, fifth, or more genders; while the gender binary that exists in most Western societies is a relatively new construct (9). “Cisgender” people have gender identities that correspond with the sex they were assigned at birth. “Gender diversity” includes individuals who identify as neither a cisgender man nor a cisgender woman (e.g., transgender, two-spirit, non-binary, genderqueer, gender non-conforming, agender, and those who are fluid in their gender identities). Researchers estimate that about 0.5–3% of the U.S. population identify as TGD (10). Due to gender being defined federally as either “male” or “female,” government surveys (e.g., U.S. Census) do not ask about TGD identities. Unfortunately, this practice is the norm in clinical research as well. The PTSD Trials Standardized Data Repository (PTSD-Repository) (11)—a resource containing interactive data from over 389 published randomized controlled clinical trials of PTSD treatment—does not include a single TGD clinical trial participant. The PTSD-Repository reports that 69% of trials included both men and women, 13% only men, 12% only women, and 6% of studies did not include gender information for participants. Thus, despite experiencing a disproportionate prevalence of PTSD, TGD people have historically not been explicitly represented in PTSD intervention research.

3,4-Methylenedioxymethamphetamine (MDMA)-assisted psychotherapy is an innovative experimental treatment for PTSD with promising results from Phase 2 (12) and Phase 3 clinical trials (13). A recent Phase 3 clinical trial of MDMA-assisted psychotherapy for PTSD included assessment of gender identity beyond binary male and female genders. In the demographics table, Mitchell et al. (13) reported “sex assigned at birth” for the 90 participants in the study (rather than traditionally reporting “gender”) and included in a footnote: “Two participants included in the assigned female at birth MDMA group identified their gender as non-binary.” It can be assumed that the other 88 participants' identified genders matched their sex assigned at birth (i.e., cisgender vs. transgender), though this is not explicitly stated. The inclusion of measurements that identify TGD people in this trial represents a positive step toward addressing the noted lack of gender diversity among participants in MDMA-assisted psychotherapy clinical research (14) and the broader fields of psychedelic research (15) and PTSD intervention research (11).

As noted by recent psychedelic research focused on the inclusion of people of color (14–16), traditional research protocols and outcomes may not generalize to all cultural groups. Providers of MDMA-assisted psychotherapy must be particularly attentive to the impact of power dynamics; transference and countertransference can be amplified, and participants may be especially open to suggestion, manipulation, and exploitation. Therefore, among people affected by significant structural vulnerabilities and health disparities, there is a unique need for increased attention to safety and consent, cultural context, trauma themes, symptom manifestation, resilience factors, and culturally adapting protocols (17).

For studies involving participants affected by structural vulnerabilities, the National Bioethics Advisory Commission recommends including the community at various points in the research process—particularly during the study planning phase (18). To achieve this, we have conducted a qualitative study with TGD participants as a formal means of initial inquiry. The aims of this study include: (1) to characterize TGD experiences with trauma-related mental health care, (2) to assess openness of TGD people to participate in experimental trauma-related mental health intervention research, specifically MDMA-assisted psychotherapy, and (3) to gather specific feedback on protocol design and clinical practice of MDMA-assisted psychotherapy for TGD people.

## Methods

### Study participants

We conducted three 90-min virtual focus group discussions (FGDs) in February and March 2021 using WebEx video conferencing. Focus group discussion participants were recruited from a medical university's Transgender Health Program, a Veterans Affairs Sexual Orientation and Gender Identity Advisory Group, and other community organizations focused on providing services and community for TGD individuals. To limit selection bias, we did not mention MDMA or MDMA-assisted psychotherapy in recruitment materials. Participants were compensated $50 upon completion of the FGD. This study was conducted in accordance with the Declaration of Helsinki and was approved jointly by the Oregon Health and Science University and the Veterans Affairs Portland Health Care System Institutional Review Boards.

We enrolled a sample (*N* = 17) consisting of TGD people (defined on recruitment material as “neither exclusively cis-male nor exclusively cis-female”). All had previous experience receiving trauma-related mental health care. Additional inclusion criteria were: (a) age ≥18 years old, (b) currently located within the United States, (c) fluent in English, (d) able to navigate WebEx and provide safety information (e.g., emergency contact information, physical location during the virtual FGD), and (e) available during one of the three scheduled FGDs. See Table 1.

**Table 1:** Demographics.

	**Mean (SD)**
**Age (years)**	35.4 (10.9)
	***n*** **(%)**
**Sample size**	17 (100)
**Gender**	
Genderqueer	1 (5.9)
Non-binary	8 (47.1)
Trans female	3 (17.7)
Trans male	4 (23.5)
Tumtum	1 (5.9)
**Race**	
American Indian	1 (5.9)
Asian	1 (5.9)
Black	2 (11.8)
White	13 (76.5)
**Hispanic ethnicity**	1 (5.9)
**Veteran**	3 (17.7)
**Education**	
High school graduate	5 (29.4)
Trade school/Some college	10 (58.8)
Bachelor's degree	3 (17.7)
Masters/Postgraduate degree	3 (17.7)
**Occupation**	
Part-time job	1 (5.9)
Self-employed	3 (17.6)
Student	5 (29.4)
Unemployed	8 (47.1)
**Disability**	5 (29.4)
**Living situation**	
Alone	2 (11.8)
With parents	2 (11.8)
With partner	6 (35.3)
With roommates	7 (41.2)
**In a primary relationship**	10 (58.8)

### Data collection

Participants received an informed consent form via email and discussed this with a researcher virtually. Verbal informed consent was videorecorded in lieu of written consent. After providing informed consent, individual participants completed a structured screening interview over WebEx. Eligible participants were then enrolled, completed a preliminary survey to assess experiences with previous trauma-related mental health care, and were scheduled for a FGD.

Both FGD co-facilitators were TGD-identified and had specialized training in trauma-informed care, gender and sexual minority issues, and MDMA-assisted psychotherapy for PTSD. Facilitators revealed their gender identities and pronouns to participants in the introduction portion of each FGD. The research team developed a semi-structured FGD guide, which started with a description of FGD ground rules and an opportunity for the participants to introduce themselves. The co-facilitators then facilitated discussion around three main topics: (1) experiences with trauma-related mental health care, (2) existing participant knowledge of MDMA-assisted psychotherapy for PTSD and potential interest in participating in research, and (3) protocol considerations for MDMA-assisted psychotherapy specifically with TGD participants. Between topics 2 and 3, the participants were given a brief, general description of the MDMA-assisted psychotherapy protocol from previous and ongoing clinical trials (13).

The day following each FGD, participants were sent a follow-up questionnaire by email. The questionnaire gathered standardized information related to interest participating in research in general as well as MDMA-assisted psychotherapy research. Lastly, the questionnaire had an open-ended space for any additional feedback that the participant would like to provide on the FGD topics.

### Analysis

Focus group discussions were recorded, transcribed verbatim, and deidentified. Transcripts were uploaded and coded using Taguette. Data analysis was conducted through identification of recurrent patterns and themes following Crabtree and Miller's five steps in qualitative data analysis, or the “interpretive process” (19). These steps are: (i) describing, (ii) organizing, (iii) connecting, (iv) corroborating, and (v) representing. Thematic analysis is an iterative process and requires frequent examination of the goals and aims of the research study. During data immersion, team members read transcripts and noted follow-up probes for later FGDs. A preliminary codebook was developed after the first FGD, which was revised as needed between FGDs to improve code definitions and include any additional themes that emerged. Three independent reviewers identified main and secondary themes.

## Results

### Previous trauma-related mental health treatment

#### Quantitative data

All participants had participated in trauma-related psychotherapy. 88.2% of the participants had taken prescribed medication for the treatment of PTSD. See Table 2 for a breakdown of psychotherapy modalities and number of previous PTSD medication trials lasting at least 3 months. Participants were also asked the following questions: 1) “How effective do you feel your previous trauma treatment was?,” 2) “How bonded did you feel to your previous trauma treatment provider(s)?,” and 3) “How culturally sensitive to gender issues were your previous trauma treatment provider(s)?.” Responses were captured using a 0–10 Likert scale (Table 2).

**Table 2:** Previous trauma treatment.

	***n* (%)**
**Previous psychotherapy modalities**
Psychodynamic	17 (100)
Cognitive behavioral therapy	15 (88.2)
EMDR	7 (41.2)
Dialectical behavior therapy	6 (35.3)
Group therapy	6 (35.3)
Internal family systems	3 (17.6)
Somatic experiencing	2 (11.8)
Equine therapy	1 (5.9)
Prolonged exposure	1 (5.9)
**Medication trials** **≥3 months**
0	2 (11.8)
1	5 (29.4)
2	4 (23.5)
3+	6 (35.3)
	**Mean (SD)**
Self-reported effectiveness of trauma treatment(s) (0–10)	6.1 (1.7)
Perceived bond to trauma treatment provider(s) (0–10)	6.4 (2.0)
Cultural sensitivity of trauma treatment provider(s) (0–10)	5.3 (3.4)

#### Qualitative thematic analysis

Several participants reported on positive experiences, particularly with mental health care providers who were TGD themselves or had a lot of experience working with TGD populations. Although one participant noted, “*I don't mean to imply…that it's not okay to want or need a queer and trans therapist because I don't think that that's true. Obviously. But it's not as important to me as it used to be.”* (P10). Another participant reported on a positive experience with an empathic provider who had neither lived TGD experience nor particular expertise in TGD health care, distinguishing the provider from systemic issues:

*It was about someone being an ally to me in the process of moving through systems that were not inclusive. Which is not something that's always thought of as good therapy, but some of the best therapy I ever had. Feeling affirmed at someone standing up for me like that, and me not having to do it. So…and then from that, a lot of trust and a lot of beautiful conversations that helped me to work through things*. (P12).

A primary theme was the general lack of available trauma-related health care with providers who were culturally aware of TGD issues. “*You have to pay out of pocket or have a job with good insurance to get that kind of therapy.”* (P4). Often, participants had to utilize whatever trauma-related mental healthcare resources were available to them through their insurance or healthcare plan. Participants mentioned barriers to receiving trauma-related psychotherapies other than cognitive behavioral therapy (e.g., eye movement desensitization and reprocessing, internal family systems), such as lack of insurance coverage or institutional support for alternative modalities. One participant noted the high turnover within a public psychiatry clinic, “*there needs to be like, a warning, so I can prepare for that, and we can finish out our sessions properly instead of cutting me and taking progress with them.”* (P6), and the subsequent dice roll when getting matched with a new provider. Another person commented on a related issue affecting access for TGD people, “*it's a small community, and so when you seek care as a person in that community from people in the community, it's just more awkward. It's more difficult.”* (P8). The potential for dual relationships with TGD providers presents an added challenge of navigating issues with confidentiality, communication, and boundaries.

Another theme was negative experiences with trauma-related health care providers. Many participants were frustrated with having to educate providers on basic TGD terminology and concepts. “*I don't like having to explain everything ‘cause they could Google things themselves.”'* (P4). Some chose to conceal their TGD identities from providers they sensed were unfamiliar with, or even hostile toward, TGD people. Reflecting on a history of TGD people being systemically pathologized within the medical system, one participant noted:

*It seems to me that, you know, many in the mental health field still regard us as mentally ill. They don't see us as…the diversity of what human beings can be. You know, because they're so binary themselves, so they're seeing the world through a binary lens—which makes it difficult for them to empathize with those of us [who] are outside of that*. (P1).

Another participant in their late 30s described their experiences of mental health treatment during childhood and adolescence:

“*I just went through the system for a very long time with a lot of like, very cisgender, white, older, male therapists. And they were…very harmful, and sort of like dictated what was wrong with me…there just wasn't like breathing room to kind of know, or have a space in which to, to explore who I was, you know? I feel like I was really told who I was through therapy for years.”* (P11).

### MDMA-assisted psychotherapy for PTSD

#### Existing knowledge and attitudes

Participant awareness and familiarity prior to the FGD of MDMA-assisted psychotherapy for PTSD was normally distributed. Three participants reported complete unfamiliarity, ten participants reported that they had heard of it or knew a little bit, three said that they knew a lot about this modality, and one did not disclose prior knowledge or lack thereof. Initial impressions ranged from enthusiasm to concern.

One participant stated, “*I'm absolutely obsessed with this. Not just MDMA, but, like, you know, I really love the work that MAPS [Multidisciplinary Association for Psychedelic Studies] is doing. I love the research that's around it.”* (P11). A few participants described their own personal experiences with MDMA or other psychedelics, including, “*I've had a lot of my own personal experiences with psychedelics, with MDMA specifically, that have been profoundly moving and life-changing. I've been staying up on the research.”* (P8).

Others were more neutral or ambivalent:

“*So, I was just saying, yeah, after, like, years of treatment-resistance and trying all the different types of therapies, all different kinds of medications, you get to a point where you're pretty much willing to try anything.”* (P3).

“*I'm not opposed to it, like, I think it could be really helpful. I think. I'm not sure if it's something I would personally do, but I think I could see it being really beneficial for people.”* (P9).

Participants also voiced several initial concerns. Multiple participants asked if current medications would need to be stopped prior to participation in MDMA-assisted psychotherapy. Conversely, a few participants were in addiction recovery and felt this would prevent them from seeking treatment with MDMA-assisted psychotherapy. In the words of one participant:

“*For me personally, one of the best decisions in recent times is actually to make a choice for sobriety, so I think it would be hard for me to really seriously consider it because I…just don't really engage in any substances for a time already and I would kind of like to keep that going, I guess.”* (P7).

Another participant added that they would still support this as an option for others:

“*I know it's something that I would never do. I'm straight edge….I do think that there's room for a lot of great and positive stuff to happen there and I want people to have access to it.”* (P10).

Lastly, concerns were voiced around vulnerable populations receiving experimental treatments, which is reflective of historical harms and ongoing mistrust in healthcare settings. One participant referenced a series of covert and illegal projects conducted between 1956 and 1971 by the United States Federal Bureau of Investigation targeting groups that were deemed to be subversive:

“*…using Veterans and at-risk groups as guinea pigs is really triggering for people of color like me, because you have things like COINTELPRO [an abbreviation derived from Counter Intelligence Program] and stuff they did in the past, and I'm like, ‘I don't know how I feel about that.”'*(P4).

#### MDMA-assisted psychotherapy protocol considerations for TGD participants

Participants offered several recommendations for future protocols of MDMA-assisted psychotherapy for TGD people. One major piece of feedback involved ensuring the screening process utilized gender-inclusive questions and phrasing (20). For example, intake coordinators should ask all potential participants their gender and pronouns. Participants noted the need for providers working with TGD participants to be explicitly gender-affirming and for the physical environment to be intentionally designed to avoid implicit cisnormative and heteronormative messaging or imagery. Overall, participants stressed a desire for a welcoming space, particularly for TGD people, who have a long history of discrimination by providers and healthcare systems. One participant mentioned:

“*…the super clinical setting is very uncomfortable for a lot of people with medical trauma, which is very common in the queer/trans community. We have all been injured at one point or another by the medical community….I don't want to be in a hospital room while I'm on MDMA. I just don't.”* (P8).

Several comments challenged the historical protocol requirement of male-female therapist dyads. While this practice may have originated to protect patients, FGD participants offered alternative suggestions to avoid harm to TGD people, such as:

“*I think that it sends a binary message. I think it should be the most qualified people that should be facilitating. It should be—if they're both males, let them be both males. If one's a trans man and one's a cis woman, let them be that. If one's a trans woman and the other one's a trans man, let them be that. You know? Whoever, whoever's qualified to run the show.”* (P1).

Another participant acknowledged more recent changes in the male–female therapy dyad:

“*I know that MAPS has edited their protocol—they used to do one male therapist and one female therapist. And having the option of having whoever you feel comfortable with—a non-binary person, a trans person…is helpful. I know that more folks are getting trained. I know that that's, you know, just been a process, but that I think would be important to me in seeking care…”* (P8).

Participants expressed a mix of interest and hesitancy regarding a group therapy protocol. While some were concerned about possibly becoming overwhelmed in a group setting, others were excited by the possibility of moving through the protocol alongside other TGD people. One participant voiced:

“*…a lot of people who go through a program like this are going to find it helpful to talk to other people with similar lived experience. And maybe not similar lived experience, but a shared experience of having taken the MDMA. I think it can be really helpful to process the things that come up with a group of people that you feel safe and comfortable with.”* (P8).

#### Post-FGD quantitative data

In a follow-up email survey, participants were asked to rate “How interested are you in participating in a research study in general (i.e., not just MDMA therapy) in which you would receive an experimental treatment for trauma-related mental health issues?” and “How interested are you in participating in research in which you would specifically receive MDMA-assisted psychotherapy that has been culturally adapted to gender diverse communities” on a scale from 0 to 10. Average respondent (*n* = 16) ratings revealed a moderate-high interest in “generally participating in trauma-related research,” *Mean(SD)*, 7.76(2.82), and similar ratings for “specifically participating in MDMA-assisted psychotherapy research,” 7.44(3.41).

## Discussion

In this study, TGD adults shared their previous experiences with trauma-related mental health care, reported their initial thoughts on participating in experimental PTSD research, and offered suggestions for tailoring existing MDMA-assisted psychotherapy protocols for TGD participants. These findings expand the existing literature on TGD peoples' experiences with healthcare providers and systems (21–23) by contributing specific information on trauma-related mental health care. The inclusion of TGD voices in early conversations about research for emerging experimental PTSD interventions allows researchers to understand the needs of the community, tailor research to meet those needs, and promotes equitable and safe access for a population disproportionately affected by trauma and PTSD who have historically been pathologized by the medical system and outwardly excluded from PTSD intervention research (11).

We have identified several key issues TGD people face when seeking and engaging in trauma-related mental health care, including: barriers to receiving adequate gender-affirming, trauma-informed mental health care; frustration with providers lacking cultural humility; and experiences overshadowed by historical medical pathologization of TGD people. Optimistically, most of the participants also described positive experiences with some providers.

The participants in our FGDs were generally curious about innovative interventions to treat post-traumatic stress, including MDMA-assisted psychotherapy. Participants voiced concerns about potential interactions of MDMA-assisted psychotherapy with other mental health treatments they may be receiving. While the decision to enroll in MDMA-assisted psychotherapy would ultimately lie with the participant, systemic medical or provider distrust may pose a barrier to critical conversations (e.g., a discussion of how to safely taper off current psychotropic regimens before receiving MDMA-assisted psychotherapy, reaching out to be screened for a clinical trial, etc.). Participants engaging in abstinence-based recovery stated that they were not likely to take MDMA—a “mind-altering substance”—as part of their PTSD treatment, despite protocols including only two or three dosing sessions. This is certainly a reasonable approach; although, recent pilot data suggests that MDMA-assisted psychotherapy could also be efficacious for the treatment of alcohol use disorder (24). Synergies between psychedelics and traditionally abstinence-based recovery programs (e.g., 12-step) have been noted for decades, and whether or not to integrate the two is still an active area of debate (25). Reservations were also expressed about vulnerable populations participating in experimental research, particularly in the context of historical harms. Equity-informed approaches recommend that providers explicitly address and invite further discussion around power, structural violence, and everyday injustices experienced by TGD people; tension and disruptions should be expected and, if meaningfully engaged with, can be productive (26).

Historically, research with psychedelics has not been exempt from contributing to medical harms against TGD and sexual minority people. For example, psychedelics have historically been paired with “conversion therapy,” based on the assumptions that TGD identities and diverse sexual orientations are mental disorders and should be “repaired” (27). Conversion therapy and other gender identity and sexual orientation change efforts have been explicitly opposed and labeled as harmful by the American Medical Association, the American Psychiatric Association, the American Psychological Association, and other professional medical associations (28–30). Researchers have called for more cultural sensitivity and affinity spaces within psychedelic medicine (15, 26, 27, 31), and organizations have been taking steps to repair historical systemic harms. For example, Multidisciplinary Association for Psychedelic Studies (MAPS)—the primary sponsor to-date for MDMA-assisted psychotherapy research—has provided 38 scholarships to TGD practitioners (and 125 for LGBTQIA + practitioners) to receive training in MDMA-assisted psychotherapy through a Health Equity Scholarship (32, 33), and the Fireside Project—a psychedelic peer support hotline—launched an Equity Initiative that will create a transgender affinity peer integration service (34).

The primary clinical outcome from previous MDMA-assisted psychotherapy studies relied on the Clinician Administered PTSD Scale to diagnose PTSD in accordance with *DSM* criteria, which centers on a single traumatic event. This method has been critiqued for conceptualizing the effects of trauma as individualized pathology as well as for not accounting for repeated or chronic experiences of oppression and violence (3, 35, 36). Recent evidence shows that MDMA and other psychedelics could reduce symptoms associated with racial trauma (14, 37) and demoralization among gay male long-term AIDS survivors (38). Similarly, formal assessment of the effect of MDMA or MDMA-assisted psychotherapy on gender minority stress and resilience is warranted (39). Gender minority stress results from gender-related discrimination, rejection, victimization, and non-affirmation and is associated with internalized transphobia, concealment of identity, negative expectations of future events, isolation, and higher rates of psychiatric comorbidities (39–41). 3,4-Methylenedioxymethamphetamine increases empathy (42), self-compassion (43), and receptivity to positive emotion (44). Increases in the personality domain of Openness were found to moderate the effects of MDMA-assisted psychotherapy on PTSD symptom reduction (45). While access to MDMA-assisted psychotherapy for TGD people does not change ongoing societal stigma and discrimination, MDMA combined with gender-affirming care stands to positively impact aspects of gender minority resilience, such as an increased sense of pride, community connectedness, and other effective coping strategies (2, 3, 39).

The participants in this study suggested several adaptations to the MDMA-assisted psychotherapy protocol for TGD participants, including: gender-inclusive intake procedures (46), gender-affirming providers and physical space (27), diversification of care teams (15), and an option for group therapy with other TGD people (47). When working with TGD people, language is critically important to rapport building (48). Standardized screening for TGD people is a simple initial step in creating an inclusive setting. The American Psychological Association published Guidelines for Psychological Practice with Transgender and Gender Nonconforming People in 2015 (46). Rea and Wallace (26) outline the provision of equity-oriented care in psychedelic medicine, specifically, using the EQUIP Health Care approach. Core dimensions of trauma- and violence-informed care, harm reduction, and culturally safe care need to be tailored to an intervention, population, and context (e.g., MDMA-assisted psychotherapy among TGD people in a medical research setting). These adaptations can be applied to general clinical research or to affinity spaces specifically for TGD people. An ongoing community engagement process will further inform priorities for TGD people and their safety when participating in experimental PTSD research.

## Limitations

Due to timing and the nature of the COVID-19 pandemic, consenting, screening, and FGDs were held virtually. To accommodate this format, inclusion criteria reflected participant ability to adequately access and navigate WebEx, potentially introducing a selection bias toward individuals with more technology fluency and economic resources than would be necessary for an in-person FGD.

In addition to public recruitment efforts, the research team made attempts to recruit Black, Indigenous, and other People of Color by extending specific requests into professional networks; although, recruitment efforts did not extend far enough in order to ensure a racially diverse sample. Racial diversity was not reflected among the research team; thus, potential participants of color may not have seen themselves represented. Furthermore, the intersectionality of race/ethnicity, TGD identity, and PTSD may have compounded to prevent interaction with a medical research group (49). Ultimately, our findings may not adequately reflect the experiences of TGD people of color. In addition to TGD-informed design, future MDMA-assisted psychotherapy research would benefit from incorporating culturally-informed strategies for people of color (50).

Our FGDs highlighted MDMA-assisted psychotherapy, due to its position at the forefront of PTSD intervention research; however, suggestions and feedback from TGD participants may apply to other forms of trauma-related interventions as well (e.g., prolonged exposure therapy, cognitive processing therapy, eye movement desensitization and reprocessing). Further research would be required.

## Conclusion

Due to stigma and systemic discrimination, TGD people experience a disproportionately high burden of trauma and PTSD. Targeted interventions are urgently needed to address health and health care disparities among TGD people, who have historically been excluded from PTSD intervention research. By including TGD people early in the research design and planning phase, this study aims to promote equitable and safe implementation of experimental PTSD intervention research. 3,4-MDMA-assisted psychotherapy is a promising emerging area of PTSD intervention research, and most of our FGD participants expressed a strong interest in participating in such research; while a few were not interested in participating for both personal and structural reasons. Amendments to current protocols for MDMA-assisted psychotherapy—such as an explicit gender-affirming treatment approach, a setting that is inclusive and culturally safe, and diversification of co-therapy dyads—will serve to promote safe and equitable access for TGD people. Future clinical trials that routinely collect trans-inclusive gender identity data and directly recruit TGD people will advance our ability to address health disparities and boost resilience factors.

## Data availability statement

The raw data supporting the conclusions of this article will be made available by the authors, without undue reservation.

## Ethics statement

The studies involving human participants were reviewed and approved by the Oregon Health and Science University and the Veterans Affairs Portland Health Care System Institutional Review Boards. Written informed consent for participation was not required for this study in accordance with the national legislation and the institutional requirements.

## Author contributions

CS acquired funding and contributed to the conception, design, data acquisition, and data analysis. MB analyzed data. DA contributed to the design and data analysis. MC contributed to the conception, design, and data acquisition. JS contributed to the conception and design. All authors contributed to the article and approved the submitted version.

## Funding

This study was funded by the Oregon Health and Science University, Department of Psychiatry through the 'OHSU Psychiatry Department Seed Money Grant. Additional salary support for CS was provided by the Department of Veterans Affairs, Clinical Science Research and Development, Federal Award Identification Number IK2CX001495.

## Conflict of interest

Author CS is a paid clinical supervisor and trainer for MDMA-assisted psychotherapy with the MAPS Public Benefit Corporation. The remaining authors declare that the research was conducted in the absence of any commercial or financial relationships that could be construed as a potential conflict of interest. The handling editor JM declared a shared affiliation with the author JS at the time of review.

## Publisher's note

All claims expressed in this article are solely those of the authors and do not necessarily represent those of their affiliated organizations, or those of the publisher, the editors and the reviewers. Any product that may be evaluated in this article, or claim that may be made by its manufacturer, is not guaranteed or endorsed by the publisher.
